# Integration: the key to implementing the Sustainable Development Goals

**DOI:** 10.1007/s11625-016-0383-3

**Published:** 2016-07-18

**Authors:** Mark Stafford-Smith, David Griggs, Owen Gaffney, Farooq Ullah, Belinda Reyers, Norichika Kanie, Bjorn Stigson, Paul Shrivastava, Melissa Leach, Deborah O’Connell

**Affiliations:** 1 CSIRO, PO Box 1700, Canberra, ACT 2601 Australia; 2 Future Earth (FutureEarth.org), Montreal, Canada; 3 Monash University, Wellington Road, Clayton, VIC Australia; 4 Warwick University, Coventry, UK; 5 Stockholm Resilience Centre, Stockholm University, Kräftriket 2B, 106 91 Stockholm, Sweden; 6 Stakeholder Forum, Unit 60, 49 Effra Road, London, SW2 1BZ UK; 7 Keio University, 5322 Endoh, Fujisawa, Kanagawa 252-0882 Japan; 8 Stigson Partners, Herrgårdsbacken 5, 131 50 Saltsjö-Duvnäs, Sweden; 9 Future Earth, Suite 1020, 1250 Guy Street, Montreal, QC H3H 2T4 Canada; 10 Concordia University, Montreal, Canada; 11 Institute of Development Studies, University of Sussex, Brighton, BN1 9RE UK

**Keywords:** Sustainable Development Goals, Means of implementation, Integration, Trade-offs and synergies, Governance, Human well-being

## Abstract

On 25 September, 2015, world leaders met at the United Nations in New York, where they adopted the Sustainable Development Goals. These 17 goals and 169 targets set out an agenda for sustainable development for all nations that embraces economic growth, social inclusion, and environmental protection. Now, the agenda moves from agreeing the goals to implementing and ultimately achieving them. Across the goals, 42 targets focus on means of implementation, and the final goal, Goal 17, is entirely devoted to means of implementation. However, these implementation targets are largely silent about interlinkages and interdependencies among goals. This leaves open the possibility of perverse outcomes and unrealised synergies. We demonstrate that there must be greater attention on interlinkages in three areas: across *sectors* (e.g., finance, agriculture, energy, and transport), across societal *actors* (local authorities, government agencies, private sector, and civil society), and between and among low, medium and high income *countries*. Drawing on a global sustainability science and practice perspective, we provide seven recommendations to improve these interlinkages at both global and national levels, in relation to the UN’s categories of means of implementation: finance, technology, capacity building, trade, policy coherence, partnerships, and, finally, data, monitoring and accountability.

**Electronic supplementary material:**

The online version of this article (doi:10.1007/s11625-016-0383-3) contains supplementary material, which is available to authorized users.

## Introduction

Nations met in September 2015 at the UN in New York and committed to the Sustainable Development Goals (SDGs)—17 global goals with 169 targets—to be met by 2030 (UN 2015). Whatever the failings of the SDGs—the *Lancet* rather harshly described them as “fairy tales, dressed in the bureaucratese of intergovernmental narcissism, adorned with the robes of multilateral paralysis, and poisoned by the acid of nation-state failure” (Horton 2015)—getting universal agreement on a defined set of goals and targets for global sustainability and human development is a remarkable achievement. However, these universal goals, the result of what the UN has described as the largest consultation in its history, will amount to little unless governments, and many non-government actors mobilize effectively to ensure that they are actually implemented.

As a framework, the SDGs extend the previous Millennium Development Goals (MDGs) in many ways, but particularly by seeking to profoundly link the social, economical, and environmental aspects of goals. This in turn implies linking across time—ensuring that the short-term achievement of improved human well-being does not occur at the cost of undermining well-being in the long term by damaging the underpinning social and environmental capital on which our global life support system depends. How is this to be assured?

Across the 16 substantive goals, 42 targets focus on “means of implementation”, albeit somewhat unevenly (Tables 1, S1), and the final goal (17) is entirely devoted to these. Spreading implementation targets throughout the goals encourages systemic implementation. However, is this enough? We suggest not. The implementation targets are largely silent about interlinkages and interdependencies among goals, regardless of their ambition to be “universal, indivisible, and interlinked” (clause 71). This leaves open the possibility of perverse outcomes, where achieving human development in the short term may undermine the capacity of the global life support system (Griggs et al. 2013) to support these advances in human well-being in the long term; or, indeed, where environmental interventions undermine the rights and well-being of certain social groups (Leach 2015). For example, promoting increased consumption to alleviate poverty may lead to the failure of other goals, such as the sustainable management of water. Uncoordinated action may create internal conflicts, such as subsidies for both renewable and non-renewable fuel sources, or missed synergies, for example, where appropriately targeted investment in renewable energy reduces emissions, but it could also reduce pollution, improve human health, and increase equality.

**Table 1:** Count of “means of implementation” listed under each substantive goal (1–16) in the seven categories defined by SDG 17 (see Table S1 for categorisation as assessed by authors; abbreviations follow the section titles in the main text following)

SDG#	Finance	Technology	Capacity	Trade	“Systemic issues”		
					Policy	Partnerships	Data, etc.
1	1				1		
2	1			2			
3	1	1	2	1	1		
4	2		3			1	
5		1			2		
6		1	2				
7		2				1	
8	1			1	1	1	
9	1	3			1		
10	2			1	1		
11	1	1			2	1	
12		1	1		1		1
13	1		1				
14		1	1	1	1		
15	2				1	1	
16			1		2		
Total	13	11	11	6	14	5	1
17	5	3	1	3	3	2	2

We suggest that there must be greater attention on these interlinkages in three areas:

- across *sectors* (e.g., finance, agriculture, energy, technology, and transport);
- across societal *actors* (local authorities, government agencies, private sector, and civil society); and
- between and among low, medium, and high income *countries*.

Here, we draw on the global sustainability science and practice perspective represented by Future Earth^1^ to provide seven recommendations to improve these interlinkages, related to the UN’s seven categories of means of implementation in SDG 17: finance, technology, capacity building, policy coherence, partnerships, and, finally, data, monitoring and accountability. In practice, much implementation will occur at national and local levels, so we conclude by suggesting how these seven issues might be supported at that level.

### Finance: link across *sectors* and *countries* through incentives for the long-term investment in early stage market development in lower income countries, particularly for products and services that support sustainable development

If the SDGs are to succeed, they must promote an inclusive approach to growth, and mobilize innovative sources of financing while phasing out investment in unsustainable activities in all countries. In general, these aims can be promoted by approaches such as Aviva Investors’ *Six Sustainable Financing Tests* (Aviva Investors, Stakeholder Forum 2015). However, businesses from high income countries still mostly avoid investment in building the business capacity of lower income countries, even where these are politically stable. Incentivising such long-term private investment from high income countries towards lower income countries requires pools of “patient capital”—capital investment that measures returns not on a quarterly or annual basis, but rather over decades and more, mandated for lower income nations.

For example, AgDevCo is a UK-based non-profit organization that invests patient capital into early stage agribusinesses in Africa (http://www.agdevco.com/). It currently invests a pool of $100 million through locally managed subsidiaries in five countries in sub-Saharan Africa (Mozambique, Ghana, Zambia, Malawi, and Tanzania). Its projects reduce rural poverty directly and indirectly by raising agricultural productivity and incomes and creating employment opportunities for rural communities. Similar links between countries and among actors need to be promoted to create businesses focused on locally appropriate products and services for sustainable development, with potential for export. This requires incentives (e.g., tax breaks) or regulatory changes (e.g., to create “B-Corporations’—http://www.bcorporation.net) in developed countries.

### Technology: link across *actors* and *countries* by promoting an integrated global innovation system for sustainable development knowledge and for technology exchanges based on environmental, economic, and cultural affinities

In the SDGs, the technology narrative is mainly framed around transferring technologies from ‘developed’ to ‘less developed’ nations. However, these may be inappropriate and in some cases delay the creation of an equitable local economy, or suppress opportunities for lower income countries to leapfrog western development pathways that have been found wanting (e.g., Berkhout et al. 2010). Timor Leste is a case in point—it experienced major deterioration and destruction of its energy infrastructure in the period leading up to independence in 1999, so that the new government naturally prioritised getting power to its citizens. However, a recent joint report with the World Bank (Ministry of Finance and World Bank 2015) concludes that the initial decisions to invest in fossil fuel-based power stations significantly delayed access to power for many citizens, and simple changes in tariff regimes would enable greater equity and efficiency in access, as well as lower emissions. Timor Leste has many opportunities for renewable power supplies, including small-scale distributed hydropower, wind, and solar systems that may not only deliver the needed energy but also enhance self-reliance, spread risk, and allow the diffusion of locally appropriate technologies. The country has a renewables strategy and could usefully access the experience of middle and high income countries with similar environments.

More broadly, implementing the SDGs will require an agile and integrated global innovation system, consciously connecting regions across the globe, linking actors in research and society, and facilitating co-production and transfer of locally appropriate knowledge and technology. An emerging example is Brazil’s strategy for south–south technology and knowledge transfer, described by *The Economist* in 2010 as a ‘global model in waiting’^2^ based around climatic, cultural, and linguistic ties to Africa and Brazil’s world-leading technical expertise in agricultural research. The opportunity is to develop a much stronger focus on facilitating the development of sustainable development friendly technologies and processes in lower income nations, with the support of higher income nations that have most environmental and cultural affinities that might also then be a trading target for these innovations. This must occur through co-design processes—that is, ensuring that all relevant stakeholders are involved in lower and higher income countries and their business sectors, in all the stages of the activities.

### Capacity building: links across *sectors* promoted through ensuring that new technologies are used to train all sectors of society in systems approaches to global sustainability

The goal of capacity building is to provide the long-term foundation for transformation. While this is often location specific, at a fundamental level, it will require all sectors in all countries to acquire new skillsets and toolkits for sustainability; this is generally noted in target 4.7,^3^ but specifically requires training and education in systems approaches to solutions, transdisciplinary initiatives, and co-design. The world needs, as the STEPS Centre puts it (Leach et al. 2012), a new generation and category of sustainability professionals who can broker between global, national, and local issues, between research and use, and between biophysical and social aspects of sustainability; notably, this need is as acute in higher as in lower income countries. At present, university teaching tends to drive towards specialisation, whereas all universities should integrate systems thinking and global sustainability into all undergraduate courses. More postgraduate courses in global sustainability are also needed, such as the 2-year Global Masters in Development Practice managed by Columbia University, which is designed as an alternative to an MBA and is now offered at more than 22 universities in 16 countries on 6 continents (http://mdpglobal.org/faq).

However, the world is on the cusp of a revolution that will see complete global internet coverage within a decade, well within the lifetime of the SDGs. There is no longer any need to rely on retrofitting inadequate institutions for this training, as suitably motivated people will be able to access training on their mobile devices: massive open online courses (MOOCs) related to sustainability and planetary boundaries are already available from the World Bank and the Sustainable Development Solutions Network, enrolling tens of thousands of people. This development avoids costly retrofitting of training institutions and can be mobilized to reset the world views of unlimited growth and the myopic focus on GDP often held by political leaders and business executives. This revolution should also be harnessed to support greater cross fertilisation of informal learning about sustainability, for example, through communities of practice, learning by doing, and reflexive learning cultures (O’Connell et al. 2013).

### Trade: linking across *countries* and *sectors* by ensuring that trade systems at all levels promote trade in appropriate products and services for sustainable development

Current trade policies, systems, and liberalisation often work against the poor and sustainability, even though when trade systems are designed with sustainable development in mind, they can boost incomes, tackle poverty and inequality, and deliver a lasting impact (Fairtrade Foundation 2015). Delivering the SDG commitment to trade policy coherence demands review and reform of domestic policies with an impact on trade, together with new approaches and accountabilities to ensure that trade agreements—bilateral, regional, and multilateral—work to support poverty alleviation and sustainability goals. This has resulted in calls for integrated initiatives around trade, where trade, food, business, and other areas of policy align to support poverty reduction, human rights, and the environment. For example, Tipping and Wolfe (2015) outline trade-related elements ranging from improving access to markets for small-scale producers to strengthening the multilateral trading system, and include commitments to reform of perverse subsidies to agriculture, fisheries and fossil fuels, as well as ensuring that regional trade and investment agreements are coherent with sustainable development objectives.

Of course, trade rules and other ways of influencing flows and investments that affect sustainability can vary across a spectrum from formal trade agreements to standards and labels and reporting mechanisms. As an example among the latter, the Carbon Disclosure Project^4^ essentially uses investor decisions in response to different levels of disclosure to drive change and a gradually improving quality of carbon accounting. However, there is a particular opportunity: global markets are increasingly exposed to a ‘green race’ (e.g., Fankhauser et al. 2013) that will favour resource efficient, low polluting products, and services that are “sustainable development friendly”. National priorities and trade rules should explicitly favour innovation and trade in such products and services that manage the links between sectors. This is increasingly happening for well-known trade-offs, such as the need for energy efficient technologies that can deliver more energy to consumers whilst reducing greenhouse gas emissions (e.g., Rogelj et al. 2013), which can be explicitly supported by policy instruments such as renewable energy targets without trying to pick specific “winner” technologies.

However, there are many opportunities in other interactions lurking among the SDGs, such as water efficiency in industrial processing, nutrient use efficiency in small-scale agriculture, other aspects of the interactions in the food–energy–water nexus, ecosystem-based management opportunities for local livelihoods, and health benefits from reducing air pollution. Griggs et al. (2014) identify a variety of these points of creative tension, but a systematic analysis is needed, so that these can be promoted (or at least not inhibited) by world trade rules. Trade rules could also be nuanced to promote the entry of lower income countries into new markets around these types of products and services. For example, Equiterre^5^ is a Quebec-based fair trade organization that, with the help of citizens, organizations and governments, develops projects in sustainable community-supported agriculture, transportation, and fair trade in coffee. Encouraging third sector organizations committed to fair and sustainable trade can have the dual effect of increasing capacity for such trade, and delivering surveillance over unfair trade practices. This last point is important, as the reality of national and commercial interests in trade negotiations is that fair outcomes do not occur without scrutiny.

### Policy and institutional coherence: link *sectors* and *actors* through strong global and national oversight of integrated development plans

A lesson from the MDGs was that individual UN agencies took charge of individual targets and implemented them with limited regard for other (particularly environmental) targets. A significant policy innovation with the SDGs is the creation of the UN’s High-Level Political Forum (HLPF), which will meet annually at the ministerial level, and every fourth year at the heads of state level. The HLPF is tasked with ensuring the integration of the three dimensions of sustainable development in a holistic and cross-sectoral manner at all levels. It is meant to have higher authority than similar previous institutions at the UN, to coordinate, secure interlinkages, mobilize resources for implementation, and monitor progress (Bernstein et al. 2014).

At national scales, arguably the scale at which implementation and achievement of the SDGs will be most critical, the SDGs will depend upon alignment and integration between national targets, strategies, and plans for implementation, as well as with national and local delivery programs. This level is thus critical to producing true policy coherence and linkages across sectors. Policy instruments, such as national sustainable development strategies, national development plans, and green economy plans, can work to link across sectors and actors. For example, national sustainable development strategies in Finland, Germany, and Wales have adopted cross-cutting, integrated approaches (using concepts such as circular economies) to delivering sustainable development (Stakeholder Forum 2015). Similarly, national and local development plans in some countries, such as China and South Africa (Li et al. 2015), and aspects of federal planning in the United States (Schaefer et al. 2015), are transcending their typical focus on economic development to open up opportunities for cross-sectoral engagement and implementation, linking areas such as water, soil, and extreme events, and biodiversity conservation. Such planning approaches need to become universal.

Even if plans are well integrated, they may be weakly implemented. Integrated implementation can be facilitated by institutions such as National Planning Commissions which bring together public and private actors, as well as civil society and academia, to forge collaborative multi-sectoral implementation. Appointing a ministry in charge of sustainable development above all other ministries, as has occurred in France and Mongolia, is another way forward; these must have the buy-in of, or influence over, the normally pre-eminent ministries, such as finance. No one actor can secure integrated implementation of the three dimensions of sustainable development; for example, business practice can be more sustainable when working in collaboration with an NGO, scientists, and the public sector (Kanie et al. 2013; Reyers et al. 2015). In particular, these arrangements need to provide the platform for a joined-up approach in civil society too, which can be as fragmented and siloed as government ministries; they also need to be echoed down to local levels to engage all of society.

### Multi-stakeholder partnerships: link across *sectors* and *actors* by encouraging widespread adoption of the SDGs as a legitimate Common Standard package

In UN-speak, ‘multi-stakeholder partnerships’ are voluntary associations between different actors, such as civil society organizations, the private sector, philanthropic organizations, and international organizations. Increasing emphasis is being placed on these partnerships to participate in developing and implementing policy for sustainable development. However, there is growing evidence that their success in developing and implementing policy hinges on engaging the *right* set of stakeholders for the issue, as well as sustained funding, and organisational learning (Pattberg and Widerberg 2015).

Another key issue identified by Pattberg and Widerberg (2015) is ‘stringent goal setting’ across actors. In this regard, the SDGs could drive innovation not only globally, but at many other levels. For example, some business groups in the Global Compact Network in Japan and elsewhere see the SDGs becoming a Common Standard for corporate social responsibility. The set of goals provides a legitimate Common Standard that could be applied globally and introduced to certification schemes that link the private sector with policy and consumers. In Australia, some local and state governments (e.g., Victorian Government 2015) believe that the SDGs provide a new, coherent framework for reporting on sustainability and human development. This kind of thinking could pave the way towards new forms of public–private partnerships (PPPs) between public and private sectors. However, these approaches will fail unless some priority is given to the integrated nature of the SDGs in these standards and partnerships, so that targets are not cherry picked but are adopted as a package—in particular, that is, so the short-term benefits for human well-being are not unduly prioritised over the long-term ones.

### Data, monitoring, and accountability: link across *countries*, *sectors*, and *actors* by developing a concise set of fully integrated indicators (‘essential sustainable development variables’)

A global indicator framework of over 230 indicators was agreed in March 2016^6^, which will be challenging to implement and monitor. Yet, our knowledge of complex systems has enabled the distillation of essential variables which capture major dimensions of change in various systems—for example, essential climate variables and essential biodiversity variables (e.g., Bojinski et al. 2014; Pereira et al. 2013), and more recently, the essential dimensions of change relevant to sustainable development, such as planetary boundaries and related frameworks (Leach et al. 2013; Steffen et al. 2015). These pave the way to identifying a set of “essential sustainable development variables” that define a core minimum set of social, environmental and economic measurements for monitoring while at the same time supporting a more integrated set of indicators for tracking and communicating progress. Such an integrated suite of indicators that link across sectors could be adapted and used by multiple actors (e.g., other UN conventions, the Common Standards discussed above), and could be aggregated across scales. However, their development and testing is a significant research undertaking which should be expedited but will not be ready in 2016.

Given significant differences in monitoring resources between countries, developing such a core minimum set of measurements for monitoring will help countries, cities, and the private sector to focus on guaranteeing this minimum data set and collection capacity. Lower income countries should be supported to collect at least this focused set of essential variables (UN SDSN 2015). This will support the tracking of cross scale, and aggregate, regional and global trends, which should be explored and promulgated through the Global Sustainable Development Report—the annual reporting mechanism to the HLPF. These would also be critical variables to encompass in modelling which will be crucial to assess whether progress is adding up to global sustainability and human well-being, to learn about how to do sustainable development and to foresight sustainable futures to backcast potential development pathways (e.g., proposed World in 2050 project^7^). This is an urgent task for the research community, such as that represented by Future Earth,^8^ in conjunction with actors across sectors and countries.

## Implementing the means of implementation

The world is moving into a new era of global governance for development and the environment, built more on trust and shared values, and on objectives, and less on legally binding frameworks. Research indicates that public commitments, either at an individual level, or national level, can drive change (Biermann and Pattberg 2008; Victor et al. 1998). However, on this scale, questions remain about how successful this can be, particularly given the influence of multinational corporations and global financial markets.

**Fig. 1:**
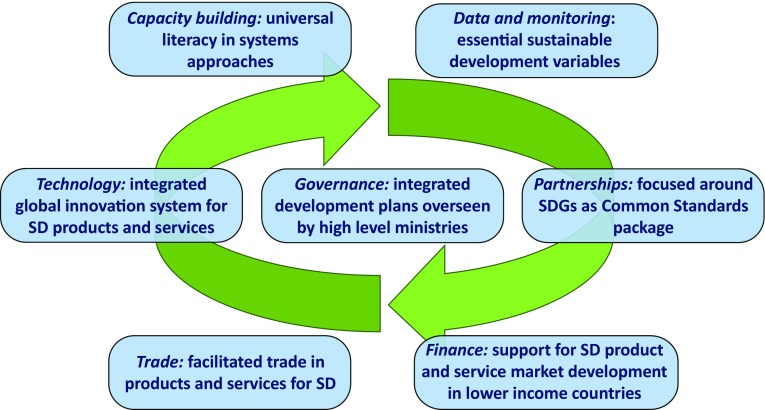
The seven categories of means of implementation in Goal 17 need to form a virtuous system, where all of them address integration issues in a coherent and self-reinforcing manner [for example, the essential variables would be applied through the Common Standards package which can then identify areas in which trade should be facilitated; finance should support technology innovation in sustainable development (SD) products in lower income countries which can then be a focus for trade]. Some key example issues are illustrated here

Therefore, globally, the means of implementation need themselves to be implemented in an integrated way. The foregoing sections identify aspects of integration in each means, but the whole set also need to be implemented in a coordinated way (Fig. 1), so that, for example, the reduced set of essential variables that might be defined under the data means of implementation, then flow through to help define the set of Standards under the partnerships means, and these in turn frame the priorities for incentives in the trade means. The agenda needs not only to pay attention to implementing the substantive goals (SDGs 1–16) in integrated ways, but also to ensuring that the means of implementation in Goal 17 and the other goals are themselves an integrated undertaking. How this can be achieved in a coordinated way is clearly a challenging question of global governance, in which the HLPF needs to address.

In practice though, each country has responsibility and sovereignty for its own development and the implementation of the SDGs, within an enabling international economic and governance environment. All member states are encouraged to develop “ambitious national responses” (UN 2015), but little guidance is provided as to how nations could keep an eye on integration whilst doing so. In the absence of this, there is a high risk that nations will ‘cherry-pick’ the goals that align with their priorities or their data collection systems, and fail to address the others that are awkward; in particular, environmental goals and targets may continue to be largely ignored or put in the too-hard basket. Instead, it is essential that nations recognise that acting well is in their own self-interest: that short term gains in their national human well-being could be readily undermined in the long term if this trade-off is not reconciled at national and global levels. The following specific seven actions, based on the foregoing discussion and universally implemented by nations within their own spheres of influence, would help to stimulate the integrated approach that sustainable development requires, in particular promoting effective linkages among *sectors*, *actors,* and *countries*, and across time frames.

- Legislative and regulatory incentives for ‘patient’ capital, particularly to be invested in lower income countries
- A partnership approach between lower and higher income *countries* to co-produce knowledge, technologies, and processes for sustainability, bearing in mind that no country is truly ‘developed’ in terms of sustainable development.
- Commitment to ensuring systems thinking is embedded in all levels of education
- Active support for trade in locally appropriate sustainable development products and services
- Integrated sustainable development plans that enforce linkages among fragmented *sectors* and promote policy coherence.
- Political leadership on sustainable development at the highest levels of government, for example, in a dedicated powerful ministry or at a supra-ministerial level, such as the executive branch
- Integrated SDG indicators supported by “essential SD variables” as a common reporting standard that encourages or requires *actors* to work together.

## Conclusion

The world has rightly paid attention to achieving an integrated agenda in the SDGs, however imperfect this achievement may have been in this first iteration of such a transformative agenda. However, this effort has focused particularly on integration among the substantive goals and targets. Vital as this is, here, we focus on the need for similar attention to obtaining a systems view and integrated approach to the means of implementation, scattered in an imbalanced way (Table 1) through all the goals and specifically in Goal 17.

In each category of means identified in Goal 17, it is possible to see how an approach could integrate and coordinate, or silo and fragment, just as is the case among the substantive targets. Here, we have presented the case for integrative thinking in the means of implementation themselves, and provided examples of how this could be achieved at global and national levels (Table 2). In the end, it is up to nations to implement the SDGs with suitable attention to local circumstances; however, there are some key actions that all nations could undertake in their own ways which would help ensure that implementation is coordinated, and provide a far greater chance of success in the lofty and vital ambitions of the post-2015 agenda.

**Table 2:** Summary of key “means of implementation” and related recommendations

Means of implementation	Key challenge	Key links needed	Recommendations	
			Globally	Nationally
1. Finance	Private sector fails to invest in sustainable development long term, especially in lower income countries	Sectors, countries	Establish incentives for long-term investment in early stage market development in lower income countries, particularly for products and services that support sustainable development	Legislative and regulatory incentives for ‘patient’ capital, particularly to be invested in lower income countries
2. Technology	Fixation on transferring technologies from ‘developed’ to ‘less developed’ countries	Actors, countries	Promote an integrated global innovation system for sustainable development knowledge and for technology exchanges based on environmental, economic, and cultural affinities	A partnership approach between lower and higher income *countries* to co-produce knowledge, technologies and processes for sustainability
3. Capacity building	Lack of deep understanding of systems and narrative of indefinite material growth	Sectors	Ensure that new technologies are used to train all sectors of society in systems approaches to global sustainability	Commitment to ensuring systems thinking is embedded in all the levels of education
4. Trade	Trade policies that disadvantage lower income countries and new entrants to sustainability markets	Countries, sectors	Ensure that trade systems at all levels promote trade in appropriate products and services for sustainable development	Active support for trade in locally appropriate sustainable development products and services
5. Policy and institutional coherence	Incentives that drive a silo mentality and failure to have high leadership on sustaining long-term human well-being	Sectors, actors	Strong global and national oversight of integrated development plans	Integrated sustainable development plans that enforce linkages among fragmented *sectors* and promote policy coherence. Political leadership on sustainable development at the highest levels of government, for example in a dedicated powerful ministry or the executive branch
6. Multi-stakeholder partnerships	Lack of discrimination about which stakeholders need to partner for different purposes, and a common purpose to partner for	Sectors, actors	Encourage widespread adoption of the SDGs as a legitimate Common Standard package	
7. Data, monitoring and accountability	Over-lengthy list of indicators to monitor, with inadequate focus on critical interactions	Countries, sectors, actors	Develop a concise set of fully integrated indicators (‘essential sustainable development variables’)	

## Electronic supplementary material

Below is the link to the electronic supplementary material.

Table S1 (DOCX 27 kb)

## Footnotes

1
http://www.FutureEarth.org.

2
http://www.economist.com/node/16592455 (“Speak softly and carry a blank cheque”, 15 July 2010).

3 Target 4.7: by 2030, ensure that all learners acquire the knowledge and skills needed to promote sustainable development, including, among others, through education for sustainable development and sustainable lifestyles, human rights, gender equality, promotion of a culture of peace and non-violence, global citizenship and appreciation of cultural diversity and of culture’s contribution to sustainable development.

4
https://www.cdp.net/en-US/Pages/HomePage.aspx.

5
http://www.equiterre.org/.

6
http://unstats.un.org/sdgs/files/report/2016/secretary-general-sdg-report-2016--EN.pdf.

7
http://unsdsn.org/blog/news/2015/03/13/the-world-in-2050-pathways-towards-a-sustainable-future/.

8
http://www.FutureEarth.org.
