# Supplementary material for: Integration: the key to implementing the Sustainable Development Goals
Source: Sustain Sci. 2016 Jul 18;12(6):911–9. doi: 10.1007/s11625-016-0383-3 (PMC6086249; doi:10.1007/s11625-016-0383-3)
Supplement: Supplementary file 1 — Table S1 (DOCX 27 kb) [file 11625_2016_383_MOESM1_ESM.docx]

Annex A – proposed on-line material

Table S1: The means of implementation included in the first 16 SDGs classified by the 7 categories in SDG 17 (source: authors’ appraisal). “Means of Implementation” categories (based on SDG 17) are: Finance, Technology, Capacity (=Capacity-building), Trade, Policy (=Policy and institutional coherence), Partnerships (=Multi-stakeholder partnerships), Data (=Data, monitoring and accountability) – some relate to multiple aspects, and we record what seem to us to be the 2 most important.

| **Goal and target** | **Text** | **Means of Implementation category** |
| --- | --- | --- |
| 1.a | Ensure significant mobilization of resources from a variety of sources, including through enhanced development cooperation, in order to provide adequate and predictable means for developing countries, in particular least developed countries, to implement programmes and policies to end poverty in all its dimensions | Finance |
| 1.b | Create sound policy frameworks at the national, regional and internationallevels, based on pro-poor and gender-sensitive development strategies, to support accelerated investment in poverty eradication actions | Policy |
| 2.a | Increase investment, including through enhanced international cooperation, inrural infrastructure, agricultural research and extension services, technologydevelopment and plant and livestock gene banks in order to enhance agricultural productive capacity in developing countries, in particular least developed countries | Finance |
| 2.b | Correct and prevent trade restrictions and distortions in world agricultural  markets, including through the parallel elimination of all forms of agriculturalexport subsidies and all export measures with equivalent effect, in accordance with the mandate of the Doha Development Round | Trade |
| 2.c | Adopt measures to ensure the proper functioning of food commodity marketsand their derivatives and facilitate timely access to market information, including on food reserves, in order to help limit extreme food price volatility | Trade |
| 3.a | Strengthen the implementation of the World Health Organization FrameworkConvention on Tobacco Control in all countries, as appropriate | Policy |
| 3.b | Support the research and development of vaccines and medicines for the communicable and non-communicable diseases that primarily affect developing countries, provide access to affordable essential medicines and vaccines, in accordance with the Doha Declaration on the TRIPS Agreement and Public Health, which affirms the right of developing countries to use to the full the provisions in the Agreement on Trade-Related Aspects of Intellectual Property Rights regarding flexibilities to protect public health, and, in particular, provide access to medicines for all. | Technology + Trade |
| 3.c | Substantially increase health financing and the recruitment, development,training and retention of the health workforce in developing countries, especially inleast developed countries and small island developing States. | Finance + Capacity |
| 3.d | Strengthen the capacity of all countries, in particular developing countries, forearly warning, risk reduction and management of national and global health risks | Capacity |
| 4.a | Build and upgrade education facilities that are child, disability and gendersensitive and provide safe, non-violent, inclusive and effective learning  environments for all | Finance + Capacity |
| 4.b | By 2020, substantially expand globally the number of scholarships available todeveloping countries, in particular least developed countries, small islanddeveloping States and African countries, for enrolment in higher education,including vocational training and information and communications technology,technical, engineering and scientific programmes, in developed countries and other developing countries | Finance + Capacity |
| 4.c | By 2030, substantially increase the supply of qualified teachers, including through international cooperation for teacher training in developing countries, especially least developed countries and small island developing States | Capacity + Partnerships |
| 5.a | Undertake reforms to give women equal rights to economic resources, as wellas access to ownership and control over land and other forms of property, financial services, inheritance and natural resources, in accordance with national laws | Policy |
| 5.b | Enhance the use of enabling technology, in particular information andcommunications technology, to promote the empowerment of women | Technology |
| 5.c | Adopt and strengthen sound policies and enforceable legislation for thepromotion of gender equality and the empowerment of all women and girls at all levels | Policy |
| 6.a | By 2030, expand international cooperation and capacity-building support todeveloping countries in water- and sanitation-related activities and programmes, including water harvesting, desalination, water efficiency, wastewater treatment, recycling and reuse technologies | Capacity + Technology |
| 6.b | Support and strengthen the participation of local communities in improvingwater and sanitation management | Capacity |
| 7.a | By 2030, enhance international cooperation to facilitate access to clean energyresearch and technology, including renewable energy, energy efficiency andadvanced and cleaner fossil-fuel technology, and promote investment in energy infrastructure and clean energy technology | Partnerships + Technology |
| 7.b | By 2030, expand infrastructure and upgrade technology for supplying modernand sustainable energy services for all in developing countries, in particular least developed countries, small island developing States and landlocked developing countries, in accordance with their respective programmes of support | Technology |
| 8.a | Increase Aid for Trade support for developing countries, in particular leastdeveloped countries, including through the Enhanced Integrated Framework forTrade-related Technical Assistance to Least Developed Countries | Finance + Trade |
| 8.b | By 2020, develop and operationalize a global strategy for youth employmentand implement the Global Jobs Pact of the International Labour Organization | Partnerships + Policy |
| 9.a | Facilitate sustainable and resilient infrastructure development in developingcountries through enhanced financial, technological and technical support to African countries, least developed countries, landlocked developing countries and small island developing States | Finance + Technology |
| 9.b | Support domestic technology development, research and innovation indeveloping countries, including by ensuring a conducive policy environment for,inter alia, industrial diversification and value addition to commodities | Policy + Technology |
| 9.c | Significantly increase access to information and communications technologyand strive to provide universal and affordable access to the Internet in leastdeveloped countries by 2020 | Technology |
| 10.a | Implement the principle of special and differential treatment for developingcountries, in particular least developed countries, in accordance with World Trade Organization agreements | Policy + Trade |
| 10.b | Encourage official development assistance and financial flows, includingforeign direct investment, to States where the need is greatest, in particular least developed countries, African countries, small island developing States and landlocked developing countries, in accordance with their national plans and programmes | Finance |
| 10.c | By 2030, reduce to less than 3 per cent the transaction costs of migrantremittances and eliminate remittance corridors with costs higher than 5 per cent | Finance |
| 11.a | Support positive economic, social and environmental links between urban,peri-urban and rural areas by strengthening national and regional development  planning | Policy |
| 11.b | By 2020, substantially increase the number of cities and human settlementsadopting and implementing integrated policies and plans towards inclusion, resource efficiency, mitigation and adaptation to climate change, resilience to disasters, and develop and implement, in line with the Sendai Framework for Disaster Risk Reduction 2015–2030, holistic disaster risk management at all levels | Partnerships + Policy |
| 11.c | Support least developed countries, including through financial and technicalassistance, in building sustainable and resilient buildings utilizing local materials | Finance + Technology |
| 12.a | Support developing countries to strengthen their scientific and technologicalcapacity to move towards more sustainable patterns of consumption and production | Technology + Capacity |
| 12.b | Develop and implement tools to monitor sustainable development impacts forsustainable tourism that creates jobs and promotes local culture and products | Data |
| 12.c | Rationalize inefficient fossil-fuel subsidies that encourage wastefulconsumption by removing market distortions, in accordance with nationalcircumstances, including by restructuring taxation and phasing out those harmful subsidies, where they exist, to reflect their environmental impacts, taking fully into account the specific needs and conditions of developing countries and minimizing the possible adverse impacts on their development in a manner that protects the poor and the affected communities | Policy |
| 13.a | Implement the commitment undertaken by developed-country parties to theUnited Nations Framework Convention on Climate Change to a goal of mobilizing jointly $100 billion annually by 2020 from all sources to address the needs of developing countries in the context of meaningful mitigation actions and transparency on implementation and fully operationalize the Green Climate Fund through its capitalization as soon as possible | Finance |
| 13.b | Promote mechanisms for raising capacity for effective climate change-relatedplanning and management in least developed countries and small island developing States, including focusing on women, youth and local and marginalized communities | Capacity |
| 14.a | Increase scientific knowledge, develop research capacity and transfer marinetechnology, taking into account the Intergovernmental Oceanographic Commission Criteria and Guidelines on the Transfer of Marine Technology, in order to improve ocean health and to enhance the contribution of marine biodiversity to the development of developing countries, in particular small island developing States and least developed countries | Capacity + Technology |
| 14.b | Provide access for small-scale artisanal fishers to marine resources and markets | Trade |
| 14.c | Enhance the conservation and sustainable use of oceans and their resources by implementing international law as reflected in the United Nations Convention on the Law of the Sea, which provides the legal framework for the conservation and sustainable use of oceans and their resources, as recalled in paragraph 158 of “The future we want” | Policy |
| 15.a | Mobilize and significantly increase financial resources from all sources toconserve and sustainably use biodiversity and ecosystems | Finance |
| 15.b | Mobilize significant resources from all sources and at all levels to financesustainable forest management and provide adequate incentives to developingcountries to advance such management, including for conservation and reforestation | Finance |
| 15.c | Enhance global support for efforts to combat poaching and trafficking ofprotected species, including by increasing the capacity of local communities topursue sustainable livelihood opportunities | Policy + Partnerships |
| 16.a | Strengthen relevant national institutions, including through internationalcooperation, for building capacity at all levels, in particular in developing countries,to prevent violence and combat terrorism and crime | Policy+ Capacity building |
| 16.b | Promote and enforce non-discriminatory laws and policies for sustainabledevelopment | Policy |
